# Ca^2+^ Permeable AMPA Receptor Induced Long-Term Potentiation Requires PI3/MAP Kinases but Not Ca/CaM-Dependent Kinase II

**DOI:** 10.1371/journal.pone.0004339

**Published:** 2009-02-03

**Authors:** Suhail Asrar, Zikai Zhou, Wei Ren, Zhengping Jia

**Affiliations:** 1 Neurosciences and Mental Health, Hospital for Sick Children, Toronto, Ontario, Canada; 2 Department of Physiology, University of Toronto, Toronto, Ontario, Canada; 3 College of Life Science, Shaanxi Normal University, Xi'an, China; L'université Pierre et Marie Curie, France

## Abstract

Ca^2+^ influx via GluR2-lacking Ca^2+^-permeable AMPA glutamate receptors (CP-AMPARs) can trigger changes in synaptic efficacy in both interneurons and principle neurons, but the underlying mechanisms remain unknown. We took advantage of genetically altered mice with no or reduced GluR2, thus allowing the expression of synaptic CP-AMPARs, to investigate the molecular signaling process during CP-AMPAR-induced synaptic plasticity at CA1 synapses in the hippocampus. Utilizing electrophysiological techniques, we demonstrated that these receptors were capable of inducing numerous forms of long-term potentiation (referred to as CP-AMPAR dependent LTP) through a number of different induction protocols, including high-frequency stimulation (HFS) and theta-burst stimulation (TBS). This included a previously undemonstrated form of protein-synthesis dependent late-LTP (L-LTP) at CA1 synapses that is NMDA-receptor independent. This form of plasticity was completely blocked by the selective CP-AMPAR inhibitor IEM-1460, and found to be dependent on postsynaptic Ca^2+^ ions through calcium chelator (BAPTA) studies. Surprisingly, Ca/CaM-dependent kinase II (CaMKII), the key protein kinase that is indispensable for NMDA-receptor dependent LTP at CA1 synapses appeared to be not required for the induction of CP-AMPAR dependent LTP due to the lack of effect of two separate pharmacological inhibitors (KN-62 and staurosporine) on this form of potentiation. Both KN-62 and staurosporine strongly inhibited NMDA-receptor dependent LTP in control studies. In contrast, inhibitors for PI3-kinase (LY294002 and wortmannin) or the MAPK cascade (PD98059 and U0126) significantly attenuated this CP-AMPAR-dependent LTP. Similarly, postsynaptic infusion of tetanus toxin (TeTx) light chain, an inhibitor of exocytosis, also had a significant inhibitory effect on this form of LTP. These results suggest that distinct synaptic signaling underlies GluR2-lacking CP-AMPAR-dependent LTP, and reinforces the recent notions that CP-AMPARs are important facilitators of synaptic plasticity in the brain.

## Introduction

The α-amino-3-hydroxy-5-methyl-4-isoxazolepropionic acid (AMPA) subtype glutamate receptors are the principal mediators of the fast excitatory synaptic transmission in the mammalian CNS and are important for the expression of various forms of long-lasting synaptic plasticity, including long-term potentition (LTP) [1]–[3]. AMPA receptors (AMPARs) are heteromeric complexes assembled from four distinct subunits (GluR1–4), of which GluR2 is particularly interesting because it dictates a number of important biophysical and biochemical properties [4]–[7]. Hence, AMPARs lacking edited GluR2 are Ca^2+^ permeable (CP-AMPAR) with higher conductance and inwardly rectifying I/V relationships.

These GluR2-lacking CP-AMPARs are widely expressed in the CNS (including interneurons, stellate and glial cells) where they can contribute to synaptic transmission and changes in synaptic efficacy [8] as well as induce multiple forms of synaptic plasticity, including LTP [9]–[15]. Subunit composition switching from GluR2-lacking to GluR2-containing AMPARs was demonstrated as fundamental to plasticity in cerebellar stellate cells [13] and the ventral tegmental area [16]. CP-AMPARs were also shown to mediate the induction and expression of LTP at neuron-glia synapses [17]. At interneuron synapses, CP-AMPARs are believed to play a crucial role in an unusual form of anti-Hebbian LTP [18].

Recent studies have also indicated that CP-AMPARs are expressed in cortical and hippocampal pyramidal neurons [8]. At developing hippocampal mossy fiber-pyramidal synapses, the selective loss of CP-AMPARs underlies a depolarization-induced form of LTD [19]. Additionally, mossy fiber-interneuron synapses were shown to demonstrate concomitant forms of LTD from either NMDARs or CP-AMPARs that were dependent on Ca^2+^ influx [20], suggesting that both types of calcium permeable receptors could work in parallel to collectively contribute to synaptic plasticity in regions where they coexist. Of particular relevance to the present study is the finding that CP-AMPARs are transiently recruited to CA1 synapses by LTP-inducing stimulations where they are involved in the consolidation of this NMDAR-dependent LTP [22]–[24], [but see 21], [25]. Finally, the expression of CP-AMPARs and the resultant Ca^2+^ influx are also associated with a number of pathophysiological states, including ischemia, epileptic seizures and drug addiction [15], [26]–[29].

Despite the importance of CP-AMPARs in synaptic regulation and pathology, the molecular processes activated by Ca^2+^ influx through these receptors is unknown. In this study, we took advantage of genetically altered mice lacking GluR2 (GluR2−/−) or having a reduced level of GluR2 (GluR2+/−) to present evidence that a distinct synaptic signaling underlies this CP-AMPAR-dependent LTP.

## Results

### CP-AMPAR-dependent LTP at CA1 synapses

**Figure 1:**
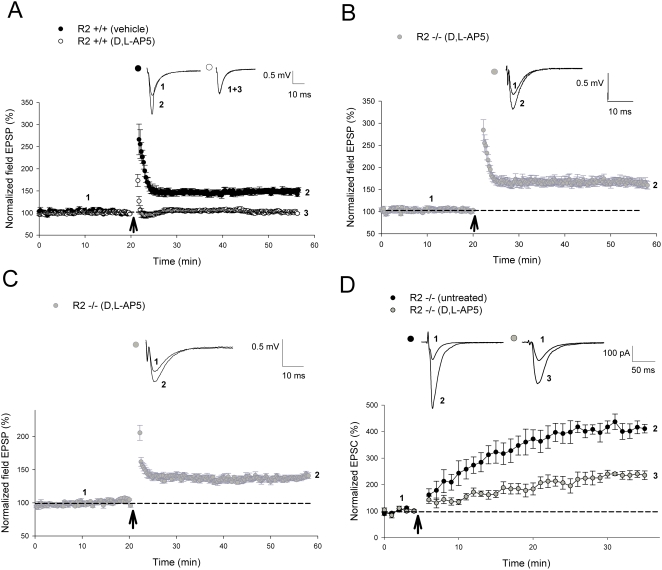
GluR2-lacking mice are capable of robust long-lasting LTP in the presence of the NMDA antagonist D,L-AP5. (A, B) D,L-AP5 completely inhibited NMDAR-dependent LTP induced by 2 trains of 100 Hz (as indicated by *arrow*) in (A) GluR2+/+slices (vehicle, n = 5; D,L-AP5, n = 5; *P*<0.001) but not CP-AMPAR-dependent LTP in (B) GluR2−/−slices (D,L-AP5, n = 6; *P*<0.001). (C, D) Robust LTP induced in GluR2−/−slices by TBS (as indicated by *arrow*) in the presence of D,L-AP5 in (C) field EPSP recordings (D,L-AP5, n = 6; *P*<0.001) and (D) whole-cell recordings (D,L-AP5, n = 6; *P*<0.001). All field EPSP recordings of CP-AMPAR-dependent LTP in GluR2 mutants involved the addition of 100 µM D,L-AP5 to perfusate 15 minutes prior to induction, lasting until 5 minutes post-induction, while all whole-cell studies of CP-AMPAR dependent LTP involved 100 µM D,L-AP5 being perfused during the entire recording period. Error bars represent SEM.

**Figure 2:**
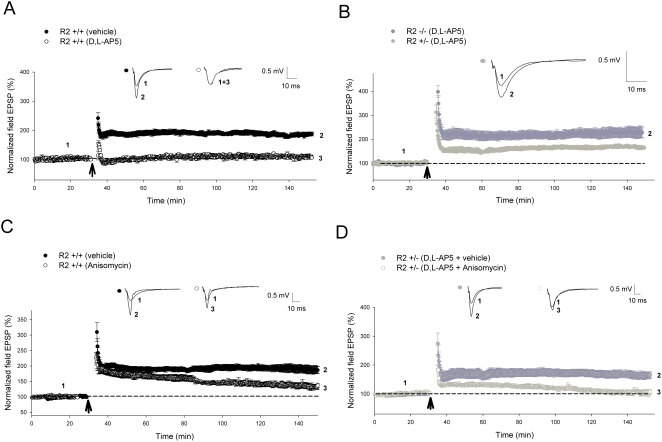
CP-AMPARs are capable of inducing long-lasting and protein-synthesis dependent forms of L-LTP. (A, B) D,L-AP5 completely blocked NMDAR-dependent L-LTP induced by 4 trains of 100 Hz (as indicated by *arrow*) in (A) GluR2+/+slices (vehicle, n = 5; D,L-AP5, n = 5; *P*<0.001) but not CP-AMPAR-dependent L-LTP in (B) GluR2−/−(D,L-AP5, n = 5; *P* = 0.002) and GluR2+/−slices (D,L-AP5, n = 5; *P*<0.001). (C, D) L-LTP induced by 4 trains of 100 Hz (as indicated by *arrow*) is dependent on protein synthesis in both (C) GluR2+/+slices (vehicle, n = 5; anisomycin n = 5; *P* = 0.006) and (D) GluR2+/−slices (vehicle, n = 5; D,L-AP5+anisomycin, n = 5; *P* = 0.002). CP-AMPAR-dependent L-LTP recordings in GluR2 mutants involved the addition of 100 µM D,L-AP5 to perfusate 15 minutes prior to induction, lasting until 5 minutes post-induction. 25 µM anisomysin was added to perfusate 15–20 minutes prior to L-LTP induction and washed away 5 minutes post-induction. Error bars represent SEM.

We have previously demonstrated that GluR2 mutants exhibit high Ca^2+^ permeability and inward rectification as well as an enhanced form of plasticity at CA1 synapses facilitated by Ca^2+^ influx through both NMDARs and CP-AMPARs [10], [30]. The utilization of an NMDAR antagonist such as D,L-AP5 allowed us to specifically isolate plasticity induced through CP-AMPARs, and thus investigate the molecular mechanisms underlying long-lasting synaptic increases induced by Ca^2+^ influx through these receptors. Therefore, in the present study we used hippocampal slices prepared from these mice to investigate CP-AMPAR-induced synaptic plasticity by performing both field and whole-cell patch-clamp recordings at the CA1 synapses. In wild-type animals, a brief high frequency stimulation (HFS, 2 trains of 100 Hz lasting 1 second) produced a long-lasting increase in field excitatory postsynaptic potentials (fEPSPs) that could be completely blocked by application of 100 µM D,L-AP5 (vehicle = 149±5.1%; D,L-AP5 = 103±1.6%; *P*<0.001) during the induction phase, indicating that this form of LTP was completely NMDAR-dependent (Figure 1A). In contrast, a significant amount of LTP was generated with the same induction protocol in GluR2−/−mice (166±8%; *P*<0.001) despite the presence of 100 µM D,L-AP5 (Figure 1B). To test whether this NMDAR-independent LTP could be induced by other stimulation protocols, we utilized theta burst stimulation (TBS), which is considered to be more physiologically relevant. As shown in Figure 1C,D, this protocol generated a significant amount of LTP during both extracellular (141±3.1%; *P*<0.001) and whole cell recordings (229±10.5%; *P*<0.001) in knockout slices in the presence of the NMDAR antagonist, in stark contrast to wild-type animals (Figure S1; control = 217±8.6%; D,L-AP5 = 102±14%; *P*<0.001). To determine the persistence of LTP induced through CP-AMPARs, we delivered multiple trains of HFS (4 trains of 100 Hz at 20 second intervals), which are commonly used induce a long-lasting (or a late phase) LTP (L-LTP). Utilizing this protocol, a CP-AMPAR dependent L-LTP (Figure 2B) was prominent in D,L-AP5-perfused GluR2−/−slices (228±20%; *P*<0.001). One potential problem with the above experiment was that all the AMPARs in GluR2−/−mice lack the GluR2 subunit, which may rarely occur under normal physiological or pathological conditions. GluR2−/−mice may also suffer developmental compensations that could lead to changes in neuronal signaling processes. Therefore, we utilized the GluR2+/−(heterozygous) mice, where the level of total GluR2 protein is reduced and both GluR2-containing and GluR2-lacking AMPARs are expressed at CA1 synapses. In addition, GluR2+/−mice are completely indistinguishable from the wild-type animals in growth and behavioral responses as opposed to GluR2−/−mice, which have multiple deficits [10]. As shown in Figure 2B, long-lasting L-LTP was also clearly generated in GluR2+/−mice in the presence of 100 µM D,L-AP5 (149±6.2%; *P*<0.001). This CP-AMPAR dependent L-LTP shared the characteristic dependence of longer-lasting forms of plasticity on the formation of new proteins [31], where plasticity induced in both wild-type (vehicle treated = 186±12.1%; anisomycin = 133±7.3%; *P* = 0.006) and GluR2+/−slices (D,L-AP5+vehicle = 163±10.8%; D,L-AP5+anisomycin = 106±7.3%; *P* = 0.002) was significantly reduced (Figure 2C,D) under the administration of the protein synthesis inhibitor anisomycin (25 µM). These results indicate that CP-AMPARs can induce various types of long-lasting synaptic plasticity at CA1 synapses, including a previously undemonstrated form of protein synthesis-dependent L-LTP that is NMDAR-independent.

### Induction of CP-AMPAR-dependent plasticity exclusively requires CP-AMPARs

**Figure 3:**
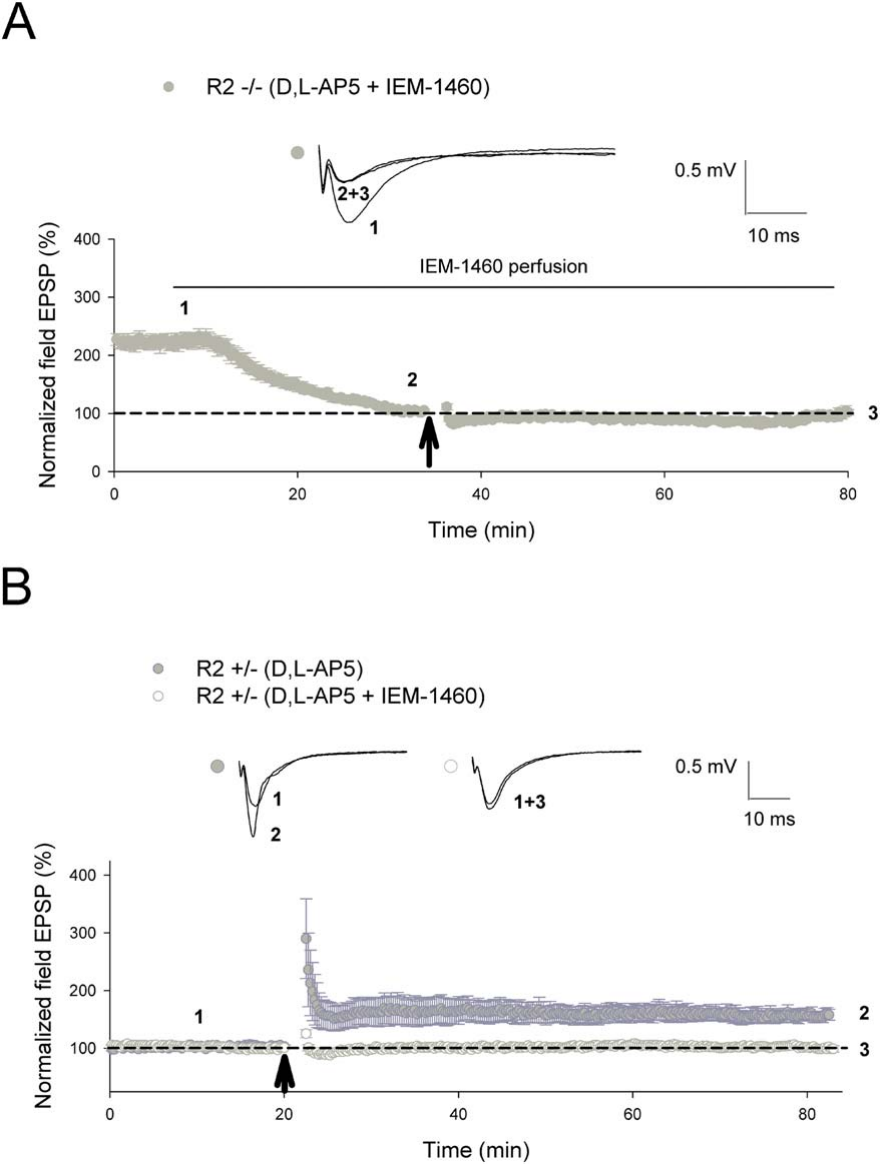
Induction of CP-AMPAR-dependent plasticity is blocked by the CP-AMPAR inhibitor IEM-1460. (A, B) Administration of IEM-1460 in (A) GluR2−/−slices significantly reduced basal synaptic response as well as completely attenuated CP-AMPAR-dependent LTP (D,L-AP5+IEM-1460, n = 6; *P* = 0.44) induced by 2 trains of 100 Hz (as indicated by *arrow*). (B) In a similar fashion, CP-AMPAR-dependent L-LTP induced by 4 trains of 100 Hz (as indicated by *arrow*) in GluR2+/−slices was also completely blocked by IEM-1460 (D,L-AP5, n = 5; D,L-AP5+IEM-1460, n = 5; *P*<0.001). All CP-AMPAR-dependent LTP field EPSP studies in GluR2 mutants involved adding 100 µM D,L-AP5 to ACSF perfusate 15 minutes prior to induction until 5 minutes post-induction. 100 µM IEM-1460 was added to the ACSF perfusate 25 minutes prior to LTP induction in GluR2−/−slices and was present throughout the entire recording period, while 100 µM IEM-1460 was added to the ACSF perfusate 15–20 minutes prior to L-LTP induction in GluR2+/−slices up until 5 minutes post-induction. Error bars represent SEM.

To exclude the possibility that other receptor subtypes (such as high voltage activated calcium channels) may play a role in the induction of CP-AMPAR-dependent plasticity, we decided to test whether this form of potentiation was susceptible to the selective CP-AMPAR inhibitor IEM-1460 [21], [32]. Administration of 100 µM IEM-1460 significantly reduced basal transmission in GluR2−/−slices (pre-treatment = 223±10.7%; treated = 105±1.8%; *P*<0.001) and completely blocked the subsequent induction of CP-AMPAR-dependent LTP by 2 trains of 100 Hz (Figure 3A) in the presence of D,L-AP5 (D,L-AP5+IEM-1460 = 101±4.7%). Accordingly, administration of 100 µM IEM-1460 in GluR2+/−slices completely inhibited CP-AMPAR-dependent L-LTP induced by 4 trains of 100 Hz (Figure 3B) in the presence of D,L-AP5 (D,L-AP5 = 157±10.1%; D,L-AP5+IEM-1460 = 102±3.7%; *P*<0.001). These results confirm that CP-AMPAR-dependent plasticity is induced exclusively through CP-AMPARs in our GluR2 knockout mouse model.

### Requirement of calcium ions in CP-AMPAR-dependent LTP

**Figure 4:**
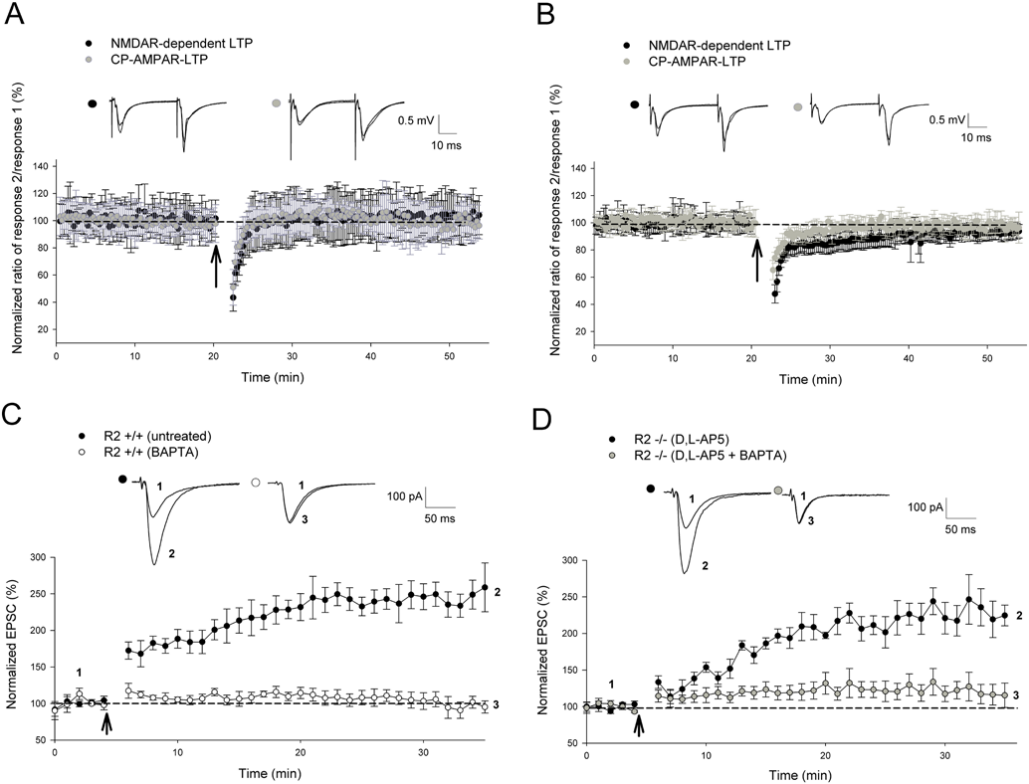
Plasticity induced through CP-AMPARs is completely dependent on Ca^2+^ influx. (A, B) Paired pulse facilitation (PPF) revealed no significant difference in presynaptic involvement in CP-AMPAR-dependent plasticity induced by 2 trains of 100 Hz (as indicated by *arrow*) in (A) GluR2−/−slices (D,L-AP5, n = 3; *P* = 0.91) and by 4 trains of 100 Hz (as indicated by *arrow*) in (B) GluR2+/−slices (D,L-AP5, n = 5; *P* = 0.97) in a manner similar to NMDAR-dependent potentiation induced in wild-type controls. (C, D) Potentiation induced by TBS (as indicated by *arrow*) in whole cell recordings (as indicated by *arrow*) is completely dependent on Ca^2+^ influx in both NMDAR-dependent LTP in (C) GluR2+/+slices (untreated, n = 5; BAPTA, n = 5; *P*<0.001) and CP-AMPAR-dependent LTP in (D) GluR2−/−slices (D,L-AP5, n = 6 ; D,L-AP5+BAPTA, n = 8; *P* = 0.001). CP-AMPAR-dependent LTP field EPSP studies in GluR2 mutants involved adding 100 µM D,L-AP5 to ACSF perfusate 15 minutes prior to induction until 5 minutes post-induction. Whole-cell recordings of CP-AMPAR dependent LTP involved the presence of 100 µM D,L-AP5 throughout the recording period. 30 mM BAPTA was included in the intracellular solution for Ca^2+^ studies. Error bars represent SEM.

To investigate the mechanisms underlying this CP-AMPAR-dependent form of potentiation, we compared paired pulse facilitation (PPF) before and during 2 trains of 100 Hz LTP (GluR2−/−with D,L-AP5; *P* = 0.91) and 4 trains of 100 Hz L-LTP (GluR2+/−with D,L-AP5; *P* = 0.97), but found no significant differences (Figure 4A,B), suggesting that presynaptic involvement was not altered following the induction of this plasticity. Therefore, we concentrated our analyses on postsynaptic mechanisms. To test whether postsynaptic calcium ions are important, we performed whole-cell patch-clamp recordings with or without the high affinity Ca^2+^ chelator BAPTA (30 mM) in the intracellular solution. As shown in Figure 4D, TBS induced a persistent increase in the amplitude of excitatory postsynaptic currents (EPSCs) that could last during the entire recording period. However, in the presence of BAPTA, the CP-AMPAR dependent LTP was completely blocked (D,L-AP5 = 228±22.7%; D,L-AP5+BAPTA = 120±13.9%; *P* = 0.001). These results indicate that Ca^2+^ ions in the postsynaptic neurons are crucial triggers for CP-AMPAR-dependent LTP, similar to their role in traditional NMDAR-dependent forms of plasticity (Figure 4C; control = 236±20.8%; BAPTA = 95±9.5%; *P*<0.001).

### Independence of Ca/CaM-dependent kinase II (CaMKII)

**Figure 5:**
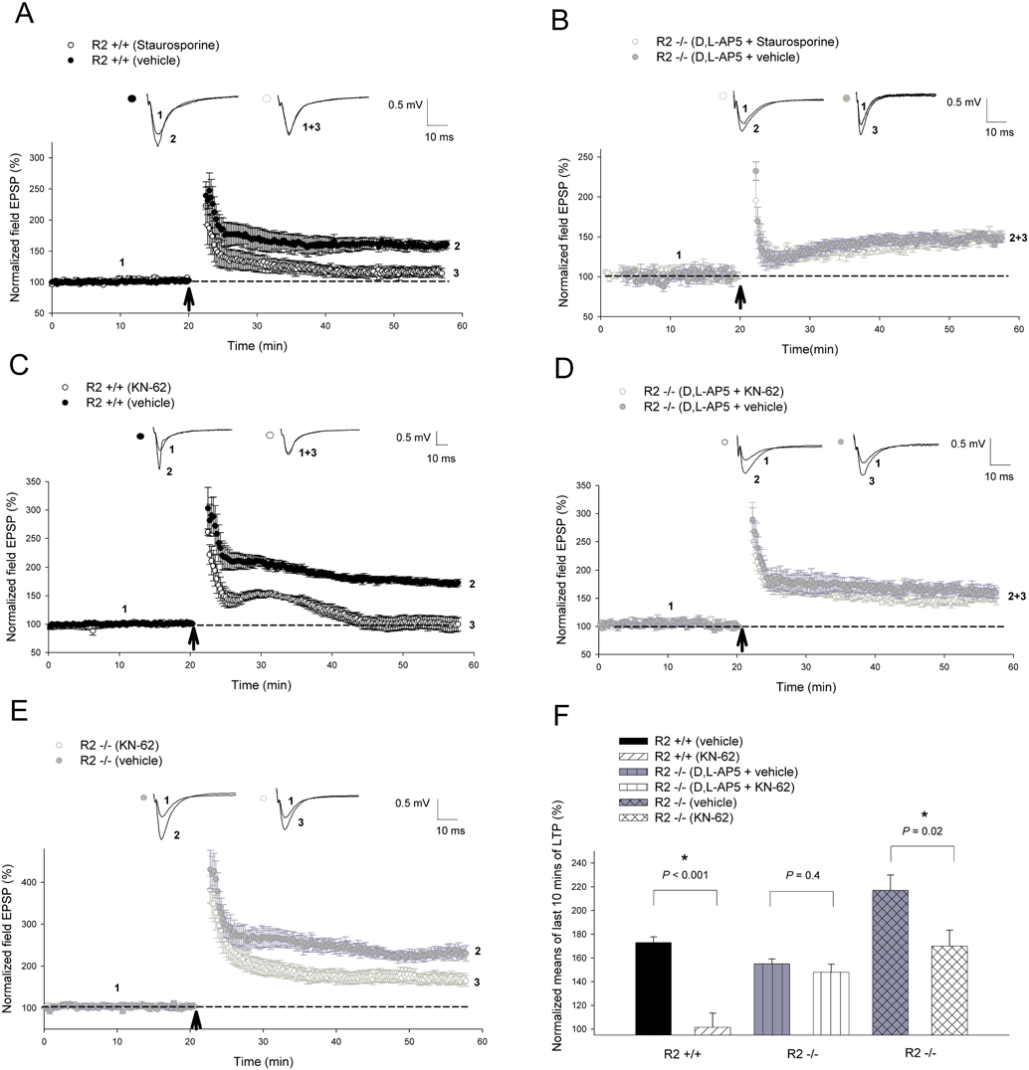
CaMKII is not involved in long-term potentiation induced through CP-AMPARs. (A, B) Contrasting effects of the broad spectrum inhibitor staurosporine where NMDAR-dependent LTP induced by 2 trains of 100 Hz (as indicated by *arrow*) was significantly attenuated in (A) GluR2+/+slices (vehicle, n = 6; staurosporine, n = 5; *P* = 0.002) but not CP-AMPAR-dependent LTP in (B) GluR2−/−slices (vehicle, n = 5; D,L-AP5+staurosporine, n = 5; *P* = 0.97). (C, D) Contrasting effects of the CaMKII-specific inhibitor KN-62 where LTP induced by 2 trains of 100 Hz (as indicated by *arrow*) was completely blocked in (C) GluR2+/+slices (vehicle, n = 5; KN-62, n = 5; *P*<0.001) but not in (D) GluR2−/−slices (vehicle, n = 5; D,L-AP5+KN-62, n = 5; *P* = 0.42). (E) However, KN-62 significantly inhibited LTP induced in GluR2−/−slices (vehicle = 6; KN-62 = 5; *P* = 0.02) in the absence of D,L-AP5. (F) Summary graph of the means of the last 10 minutes of potentiation seen in KN-62 treatment studies. All CP-AMPAR-dependent LTP recordings in GluR2 mutants involved the administration of 100 µM D,L-AP5 to perfusate 15 minutes prior to induction, lasting until 5 minutes post-induction. 100 nM staurosporine and 15 µM KN-62 were added to perfusate 15–20 minutes prior to LTP induction and washed away 5 minutes post-induction. Error bars represent SEM. * denotes *P*<0.05.

In NMDAR-dependent LTP at CA1 synapses, Ca^2+^ influx from NMDARs activate CaMKII to trigger a number of downstream events, including AMPAR trafficking to the synapse that ultimately result in an increase in synaptic transmission [1], [33]–[35]. Therefore, CaMKII is the key Ca^2+^-activated protein kinase indispensable for the induction of NMDAR-dependent LTP. To test whether CaMKII also plays a role in CP-AMPAR-dependent LTP induced by 2 trains of 100 Hz, we first utilized the broad spectrum CaMKII inhibitor staurosporine [36], [37]. Administration of 100 nM staurosporine drastically reduced NMDAR-dependent LTP (Figure 5A) in the wild-type animals (vehicle treated = 160±6.9%; staurosporine = 116±7.5%; P = 0.002), but surprisingly had no effect on the amount of CP-AMPAR dependent LTP (Figure 5B) induced in the presence of D,L-AP5 (D,L-AP5+vehicle = 149±6.4%; D,L-AP5+staurosporine = 148±3; *P* = 0.97). To confirm these findings, we performed further investigations by including the CaMKII specific inhibitor KN-62 in the perfusion solution. Accordingly, we found that KN-62 (15 µM) also had no effect on CP-AMPAR-induced LTP in GluR2−/−mice. As shown in Figure 5D, the magnitude of LTP was indistinguishable with or without KN-62 (D,L-AP5+vehicle = 155±4.2%; D,L-AP5+KN-62 = 148±6.8%; *P* = 0.42). The lack of KN-62 effect on CP-AMPAR-dependent LTP was not due to the ineffectiveness of the drug because it could effectively block NMDAR-dependent LTP (Figure 5C) in wild-type animals (vehicle treated = 174±4.8%; KN-62 = 102±12.1%; *P*<0.001) and also significantly inhibited the enhanced LTP in GluR2−/−mice (Figure 5E) in the absence of D,L-AP5 (vehicle treated = 221±12.4%; KN-62 = 170±13.4%; *P* = 0.02). These results indicate that CaMKII is not required for CP-AMPAR-dependent LTP, and suggest that a distinct synaptic signaling cascade must be operating during this form of LTP.

### Requirement for mitogen-activated kinase (MAPK) cascade and phosphoinositide 3-kinase (PI3-kinase)

**Figure 6:**
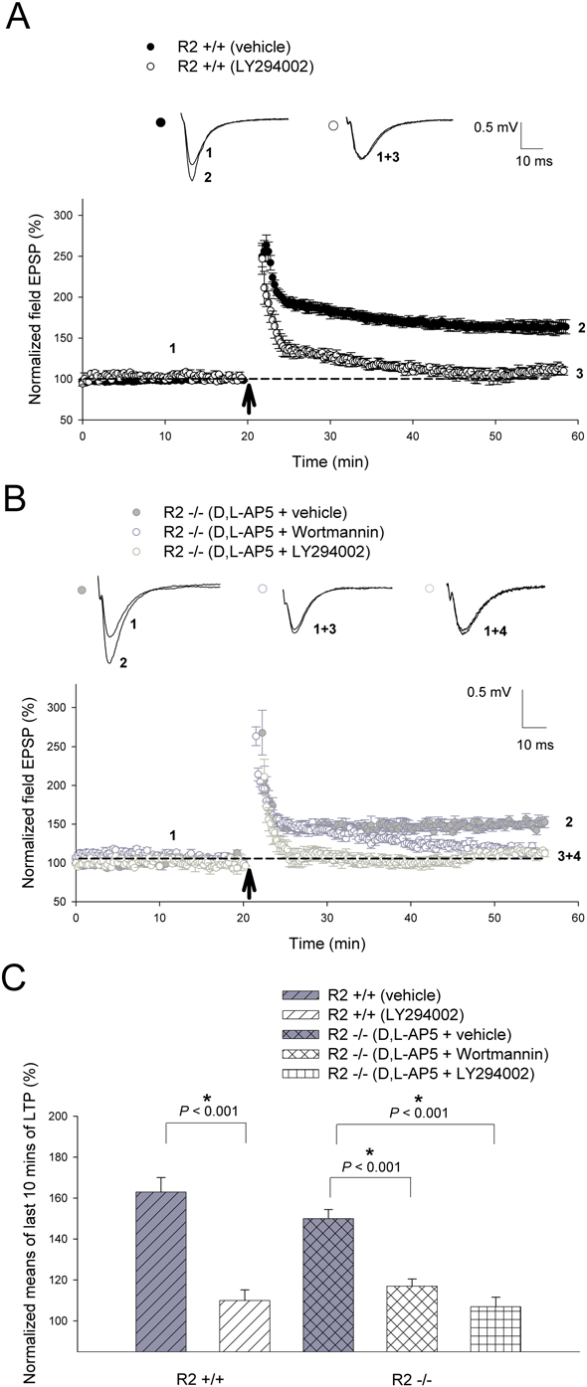
PI3-kinase is required for CP-AMPAR-dependent long-term potentiation. (A) The specific PI3-kinase inhibitor LY294002 significantly attenuated NMDAR-dependent LTP induced by 2 trains of 100 Hz (as indicated by *arrow*) in GluR2+/+slices (vehicle, n = 6; LY294002, n = 5; *P*<0.001). CP-AMPAR-dependent LTP elicited by 2 trains of 100 Hz (as indicated by *arrow*) in (B) GluR2−/−slices was also strongly suppressed in the presence of the structurally unrelated PI3K inhibitors LY294002 (vehicle, n = 5; D,L-AP5+LY294002, n = 5; *P*<0.001) and wortmannin (vehicle, n = 5; D,L-AP5+wortmannin, n = 5; *P*<0.001). (C) Summary graph of the means of the last 10 minutes of potentiation seen in LY294002 and wortmannin treatment studies. CP-AMPAR-dependent LTP recordings in GluR2 mutants involved the addition of 100 µM D,L-AP5 to perfusate 15 minutes prior to induction, ceasing at 5 minutes post-induction. 20 µM LY294002 and 1 µM wortmannin were added to ACSF perfusate 15–20 minutes prior to LTP induction up until 5 minutes post-induction. Error bars represent SEM. * denotes *P*<0.05.

**Figure 7:**
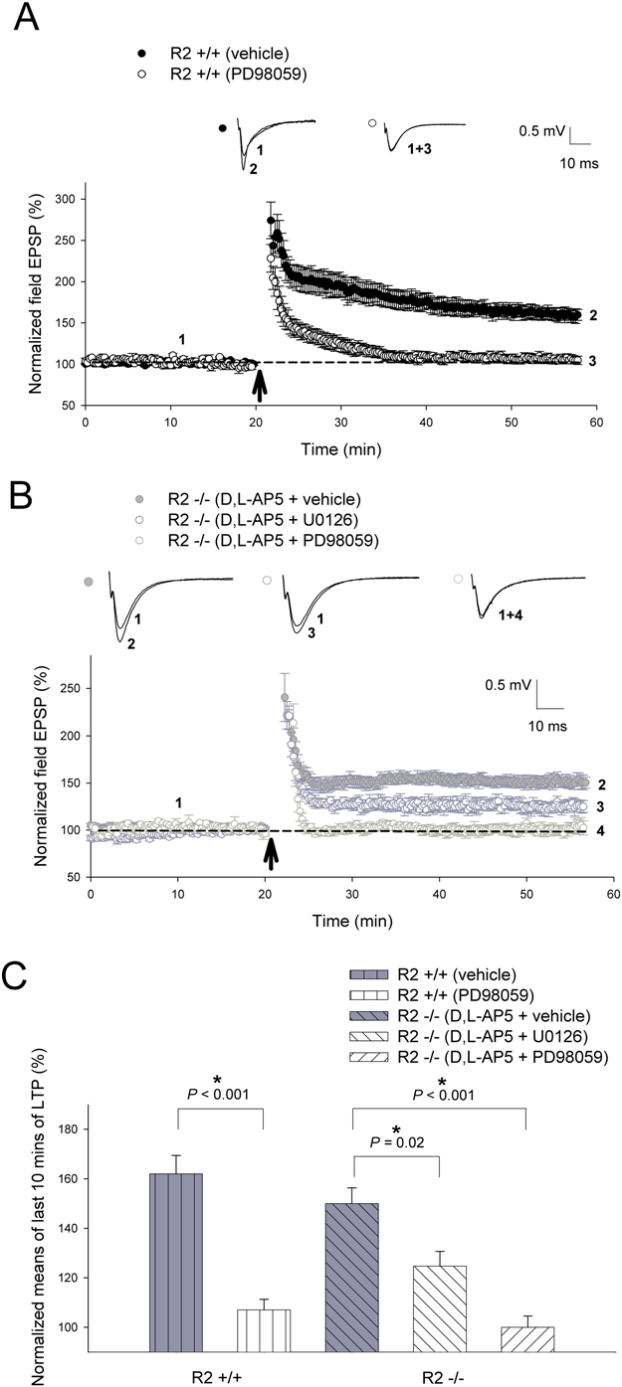
The MAPK signaling cascade plays an essential role in long-term potentiation induced through CP-AMPARs. (A) The specific MEK inhibitor PD98059 attenuated LTP elicited by 2 trains of 100 Hz (as indicated by *arrow*) in GluR2+/+slices (vehicle, n = 5; PD98059, n = 6; *P*<0.001). CP-AMPAR-dependent LTP induced by 2 trains of 100 Hz (as indicated by *arrow*) was also significantly inhibited in (B) GluR2−/−slices by the structurally unrelated MEK inhibitors PD98059 (vehicle, n = 6; D,L-AP5+PD98059, n = 5; *P*<0.001) and U0126 (vehicle, n = 6; D,L-AP5+U0126, n = 5; *P* = 0.02). (C) Summary graph of the means of the last 10 minutes of potentiation seen in PD98059 and U0126 treatment studies. CP-AMPAR-dependent LTP recordings in GluR2 mutants involved the addition of 100 µM D,L-AP5 to perfusate 15 minutes prior to induction, ceasing at 5 minutes post-induction. 50 µM PD98059 and 35 µM U0126 were added to ACSF perfusate 15–20 minutes prior to LTP induction lasting until 5 minutes post-induction. Error bars represent SEM. * denotes *P*<0.05.

Recent studies suggest that both MAPK and PI3-kinases are involved in NMDAR-dependent LTP and AMPAR trafficking in the hippocampus [38]–[44]. Therefore, we tested whether they are also important in CP-AMPAR-dependent LTP induced by 2 trains of 100 Hz by comparing the effects of MEK and PI3-kinase inhibitors. Utilization of the PI3-kinase inhibitor LY294002 (20 µM) completely blocked both NMDAR-dependent LTP (Figure 6A) in the wild-type animals (vehicle treated = 163±7%; LY294002 = 110±5.2%; *P*<0.001) and CP-AMPAR-dependent LTP (Figure 6B) in GluR2−/−mice (D,L-AP5+vehicle = 150±4.4; D,L-AP5+LY294002 = 107±4.6; *P*<0.001). Accordingly, administration of wortmannin (1 µM), another PI3-kinase inhibitor that is structurally unrelated to LY294002, also attenuated CP-AMPAR dependent LTP in knockout slices (D,L-AP5+vehicle = 150±4.4; D,L-AP5+wortmannin = 117±3.5; *P*<0.001; Figure 6B). In a similar manner, the use of MEK (MAPKK or ERK kinase) inhibitor PD98059 (50 µM) strongly suppressed LTP (Figure 7A,B) in both wild-type (vehicle treated = 162±7.4%; PD98059 = 107±4.3%; *P*<0.001) and GluR2−/−mice (D,L-AP5+vehicle = 150±6.3%; D,L-AP5+PD98059 = 100±4.6%; *P*<0.001). The involvement of the MAPK cascade in CP-AMPAR dependent plasticity was further demonstrated by the suppression of LTP in knockout slices (D,L-AP5+vehicle = 150±6.3%; D,L-AP5+U0126 = 125±6%; *P* = 0.02; Figure 7B) by the PD98059-structurally unrelated MEK inhibitor U0126 (35 µM). These results indicate that both PI3-kinase and MAPK signaling pathways are required for CP-AMPAR-dependent LTP.

### Role of MAPK in the induction of plasticity

**Figure 8:**
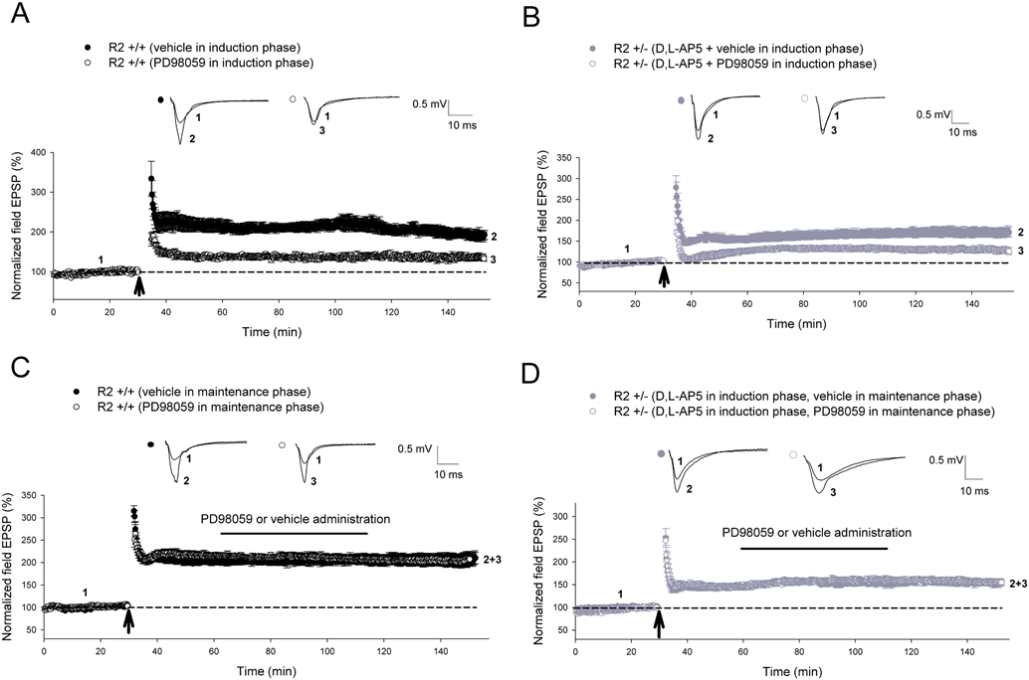
The MAPK signaling cascade plays a role in the induction but not maintenance of plasticity induced through CP-AMPARs. (A, B) Administration of the MEK inhibitor PD98059 during the induction phase of L-LTP significantly attenuated potentiation induced by 4 trains of 100 Hz (as indicated by *arrow*) during both NMDAR-dependent L-LTP in (A) GluR2+/+slices (vehicle, n = 5; PD98059, n = 6; *P* = 0.002) and CP-AMPAR-dependent L-LTP in (B) GluR2+/−slices (vehicle, n = 5; D,L-AP5+PD98059, n = 5; *P* = 0.009). (C, D) However, administration of PD98059 during the maintenance phase of L-LTP induced by 4 trains of 100 Hz (as indicated by *arrow*) had no significant effect on both (C) GluR2+/+slices (vehicle, n = 5; PD98059, n = 5; *P* = 0.9) and (D) GluR2+/−slices (vehicle, n = 5; D,L-AP5+PD98059, n = 5; *P* = 0.83). All CP-AMPAR-dependent L-LTP recordings in GluR2 mutants involved adding 100 µM D,L-AP5 to perfusate 15 minutes prior to induction until 5 minutes post-induction. For MAPK L-LTP induction studies, 50 µM PD98059 was added to ACSF perfusate 15–20 minutes prior to induction lasting until 5 minutes post-induction. For MAPK L-LTP maintenance studies, 50 µM PD98059 was added to the ACSF perfusate 20–30 minutes post-induction. Error bars represent SEM.

To further elucidate the role of the MAPK signaling cascade in CP-AMPAR dependent plasticity, we tested whether the MEK inhibitor PD98059 (50 µM) would attenuate potentiation when perfused during the maintenance phase of CP-AMPAR dependent L-LTP induced by 4 trains of 100 Hz. We first demonstrated that the administration of this inhibitor (Figure 8A) prior to and during the induction of L-LTP in wild-type slices largely inhibited potentiation (vehicle treated = 192±11.2%; PD98059 = 137±6.7%; *P* = 0.002), while having no significant effect when introduced during the maintenance phase (Figure 8C) of this NMDAR-dependent plasticity (vehicle treated = 206±12.5%; PD98059 = 204±12.7%; *P* = 0.9). In a similar manner, the presence of PD98059 in GluR2+/−slices significantly blocked CP-AMPAR dependent L-LTP in the inductory (D,L-AP5+vehicle in induction phase = 171±10%; D,L-AP5+PD98059 in induction phase = 129±6.3%; *P* = 0.009) but not the maintenance phase (D,L-AP5+vehicle in maintenance phase = 155±6.3%; D,L-AP5+PD98059 in maintenance phase = 154±5.5%; *P* = 0.83) of this form of potentiation (Figure 8B,D). These results suggest that the MAPK signaling cascade is an essential component in the induction but not maintenance of both NMDAR- and CP-AMPAR dependent forms of plasticity.

### Partial requirement of receptor trafficking

**Figure 9:**
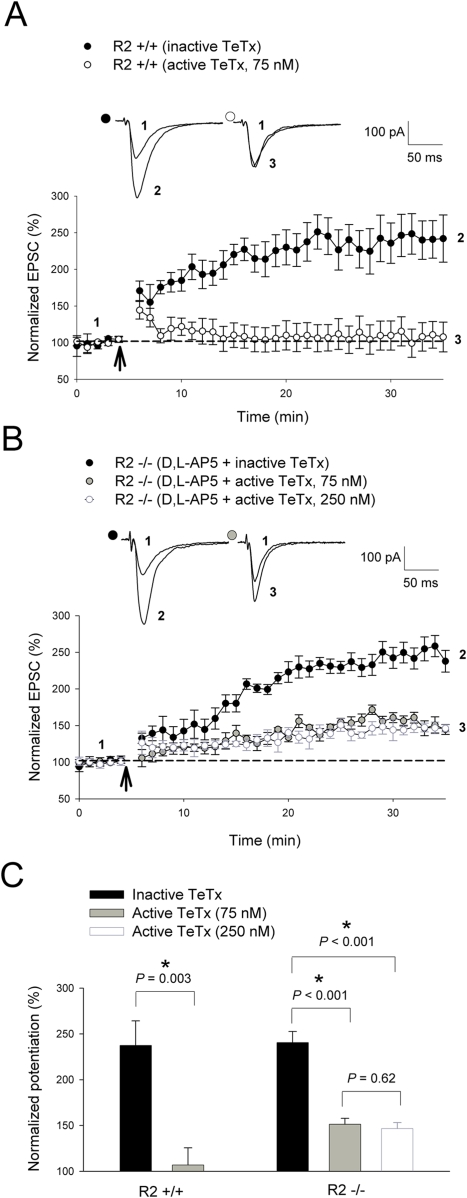
Receptor trafficking plays an important role in plasticity induced through CP-AMPARs. (A) Synaptic plasticity induced by TBS (as indicated by *arrow*) was significantly attenuated in the presence of the exocytosis-inhibiting tetanus toxin (TeTx, 75 nM) during NMDAR-dependent LTP in GluR2+/+slices (inactive toxin, n = 6; 75 nM TeTx, n = 6; *P* = 0.003). (B) CP-AMPAR-dependent LTP elicited by TBS (as indicated by *arrow*) in GluR2−/−slices was also significantly reduced in the presence of 75 nM TeTx (D,L-AP5+inactive toxin, n = 7; D,L-AP5+75 nM TeTx, n = 5; *P*<0.001) and 250 nM TeTx (D,L-AP5+inactive toxin, n = 7; D,L-AP5+250 nM TeTx, n = 5; *P*<0.001) respectively to statistically similar levels (D,L-AP5+75 nM TeTx, n = 5; D,L-AP5+250 nM TeTx, n = 5; *P* = 0.62). (C) Summary graph of the means of the last 5 minutes of potentiation seen in tetanus toxin treatment studies. Whole-cell recordings of CP-AMPAR dependent LTP involved the presence of 100 µM D,L-AP5 throughout the recording period. 75 nM and 250 nM TeTx were included in the intracellular solution for exocytosis inhibition studies. Error bars represent SEM. * denotes *P*<0.05.

Ample results have indicated that AMPAR trafficking at the synapse is critical for synaptic changes [1], [34], [35]. Thus, expression of NMDAR-dependent LTP is dependent on increased AMPAR insertion through regulated exocytosis. To determine whether this process plays a role in CP-AMPAR-dependent LTP, we examined the effect of the exocytosis-inhibiting tetanus toxin light chain fragment (TeTx) on plasticity by including the toxin in the intracellular recording solution. Consistent with previous reports [45], TeTx (75 nM) completely blocked NMDAR-dependent LTP (Inactive TeTx = 237±26.7%; active TeTx, 75 nM = 107±18.9%; *P* = 0.003) induced by TBS (Figure 9A) in the wild-type animals. In GluR2−/−slices, the magnitude of CP-AMPAR dependent TBS-LTP in the presence of D,L-AP5 was also significantly reduced (D,L-AP5+inactive TeTx = 241±12%; D,L-AP5+active TeTx, 75 nM = 151±6.4%; *P*<0.001) by 75 nM TeTx (Figure 9B). However, unlike NMDAR-dependent LTP, plasticity induced through CP-AMPARs was not completely abolished in the presence of the toxin, including when the concentration of TeTx was increased to 250 nM (D,L-AP5+active TeTx, 75 nM = 151±6.4%; D,L-AP5+active TeTx, 250 nM = 147±6.6%; *P* = 0.62; Figure 9B,C). These results suggest that exocytosis plays at least a partial role in plasticity induced through CP-AMPARs.

## Discussion

In this study, by using GluR2−/−and GluR2+/−mice, we were able to induce and investigate the underlying mechanisms of synaptic plasticity caused by CP-AMPARs in the CA1 region of the hippocampus. We demonstrated that these receptors can induce a long-lasting enhancement in synaptic strength that is Ca^2+^ dependent, but surprisingly independent of CaMKII, a crucial regulator of NMDAR-dependent LTP. Instead, this form of LTP requires both the PI3-kinase and MAPK signaling cascades, as well as exocytosis. These results indicate a unique synaptic signaling process during CP-AMPAR-induced synaptic plasticity.

We also demonstrated that a number of protocols were able to induce CP-AMPAR-dependent LTP. These include TBS (Figure 1, 4, 9) that is often used to induce NMDAR-dependent LTP at resting membrane potentials, suggesting that CP-AMPAR-dependent synaptic plasticity may occur under physiological conditions. Additionally, we also presented the first ever demonstration of the capability of CP-AMPARs to induce and sustain a longer-lasting protein synthesis dependent form of L-LTP in the CA1 region of the hippocampus, previously thought to be a hallmark of NMDARs alone.

CP-AMPAR-dependent LTP was completely blocked by postsynaptic inclusion of 30 mM BAPTA, indicating that calcium ions in the postsynaptic neurons are required (Figure 4). Consistent with this, we and others have previously shown that GluR2-lacking AMPARs exhibit significant Ca^2+^ permeability [7], [10] that is similar to that of NMDARs; therefore, the Ca^2+^ influx from these receptors is likely sufficient to trigger Ca^2+^-dependent events in the postsynaptic neurons. Ca^2+^ transients resulting from synaptically activated CP-AMPARs have also been demonstrated in the cortex and shown to amplify the transient amplitude when co-expressed with NMDARs, suggesting the presence of mechanisms that allow the proportional scaling of each receptor type independent of presynaptic miniature-release factors [46].

Since CP-AMPAR-dependent LTP was also inducible in the presence of high voltage activated calcium channel blockers [10], the most likely calcium source in this form of plasticity is the Ca^2+^ influx from CP-AMPARs. This notion was confirmed by our results showing that CP-AMPAR-dependent plasticity induced by either 2 trains or 4 trains of 100 Hz was completely blocked by the selective CP-AMPAR inhibitor IEM-1460 in GluR2−/−and GluR2+/−mutants respectively (Figure 3). In contrast to GluR2−/−animals, the same concentration of IEM-1460 (100 µM) had a small but non-significant effect on the basal synaptic response in GluR2+/−slices, suggesting that CP-AMPARs may not play an important role in basal transmission at CA1 synapses, and reinforces the idea that Ca^2+^ influx from newly recruited forms of these receptors may help consolidate previously induced plasticity in the region [22]–[24], [but see 21], [25].

Given the critical role for Ca^2+^ in CP-AMPAR-dependent LTP, it is therefore surprising to discover that CaMKII is not required for this form of LTP as the CaMKII-specific inhibitor KN-62 had no effect (Figure 5). In addition, the general kinase inhibitor staurosporine, which is also known to block the actions of PKC, PKA, and protein tyrosine kinases in addition to CaMKII [36], [37], also had no inhibitory effects on CP-AMPAR-dependent LTP. The lack of effect of these two inhibitors is not due to the ineffectiveness of the drugs because they strongly inhibited NMDAR-dependent LTP in slices prepared from wild-type littermate animals (Figure 5A,C). Additionally, the enhanced LTP (comprising of both NMDAR- and CP-AMPAR-dependent components in the absence of D,L-AP5, Figure 5E) seen in GluR2−/−mice [10] was also significantly, but not completely, attenuated in the presence of KN-62. This would suggest that the CaMKII inhibitor worked only towards blocking the NMDAR-dependent component of the enhanced LTP induced in GluR2−/−mice when D,L-AP5 was not present, while not affecting the CP-AMPAR-dependent component, corresponding with our pharmacological inhibitor results in wild-type and mutant animals respectively. It is also important to note that the expression and targeting of CaMKII is not altered in GluR2−/−or GluR2+/−mice (data not shown). These results indicate that Ca^2+^ influx from CP-AMPARs initiates a distinct synaptic signaling process that is different from the activation of NMDARs. Interestingly, CaM and CaMKII are associated with the NMDAR but not AMPAR complex [47]–[49].

In contrast to CaMKII, our results provide evidence for the involvement of the PI3-kinase (Figure 6) and MAPK signaling cascades (Figure 7) in CP-AMPAR dependent plasticity. This was accomplished through the utilization of two separate pairs of structurally unrelated inhibitors geared towards each of these kinase systems (LY294002 and wortmannin for PI3K; PD98059 and U0126 for MAPK respectively). The varying degrees of attenuation of CP-AMPAR-dependent LTP by the above-mentioned drugs was probably due to differences in concentration and the modalities of action of these individual inhibitors. It is interesting to note that CP-AMPAR dependent L-LTP was not completely inhibited by the same concentration of PD98059 (Figure 8B) that abolished CP-AMPAR dependent LTP in knockout slices (Figure 7B). This could be explained by the possibility that the stronger stimulation protocols utilized for L-LTP need a longer period of time for plasticity to completely decay, requiring an extended period of post-inductory recording. Additionally, mechanistic differences between LTP and L-LTP have been previously described [31], suggesting that the induction of the latter form of plasticity may recruit additional biochemical pathways that are distinct and insensitive to MAPK inhibitors. These notions are supported by the fact that L-LTP induced in wild-type slices was also not completely blocked by the same concentration of PD98059 in a similar time period (Figure 8A), in comparison to the total abolishment of LTP induced by 2 trains of 100 Hz in the same animals (Figure 7A).

The involvement of the PI3-kinase and MAPK cascades in this form of plasticity is of particular interest as both have also been shown to be important for NMDAR-dependent LTP [39], [40]. We therefore hypothesize that these two signaling pathways may serve as a common target for both NMDAR- and CP-AMPAR-dependent LTP. Since CP-AMPAR-dependent LTP is independent of CaMKII, our model suggests that CP-AMPARs may act downstream of NMDARs and CaMKII, consistent with the idea that CP-AMPAR-activated signaling process may serve as a mechanism for the consolidation of NMDAR-dependent LTP [22]. To support this notion, a recent study implicated another member of the CaMK family (CaMKI) in the specific synaptic recruitment of CP-AMPARs during TBS-LTP in the CA1 region [24]. Furthermore, the shared dependence of both NMDAR- and CP-AMPAR-induced types of L-LTP on new protein synthesis may suggest that Ca^2+^ influx through newly recruited GluR2-lacking receptors could also be an important facilitator of traditional longer-lasting forms of plasticity in the hippocampus.

Our results are consistent with the hypothesis that the expression mechanisms of CP-AMPAR-dependent LTP are postsynaptic. This is based on the findings that PPF does not change after LTP induction (Figure 4) and that postsynaptic injection of exocytosis-inhibiting tetanus toxin (75 nM) largely blocks this form of LTP (Figure 9). However, since the inhibiting effect of tetanus toxin is not complete even at higher concentration levels (250 nM; Figure 9B,C), other mechanisms independent of receptor trafficking may also contribute to CP-AMPAR-dependent LTP. These may include changes in receptor protein phosphorylation and subunit composition. It is known that multiple postsynaptic mechanisms exist for NMDAR-dependent LTP at CA1 synapses [35]. The requirement of AMPAR insertion for CP-AMPAR-dependent LTP is also consistent with the above model that CP-AMPAR-induced LTP is downstream of NMDAR-dependent LTP. This mechanism has been postulated to consolidate synaptic enhancement as newly inserted GluR2-lacking CP-AMPARs are gradually replaced by GluR2-containing receptors [22]–[24], a process that likely depends on exocytosis.

In summary, we have identified a unique signaling pathway underlying long-lasting synaptic enhancement triggered by Ca^2+^ influx through CP-AMPARs at CA1 synapses. We suggest that this synaptic signaling process may provide an important and general mechanism for synaptic plasticity at synapses where the expression of GluR2 is dynamically regulated under various circumstances, including development, synaptic plasticity and pathological conditions.

## Materials and Methods

### GluR2 knockout mice

GluR2 knockout mice were created and bred as previously described [10], [30]. For genotyping of GluR2 knockout mice, a standard protocol for Taqman PCR was used. A common reverse primer 5′-TCGCCCATTTTCCCATATAC-3′ and forward primers 5′-GGTTGGTCACTCACCTGCTT-3′ and 5′-TCGCCCATTTTCCCATATAC-3′ were used to detect wild-type allele and the neomycin resistance cassette in knockout mice respectively. All studies with mutant animals (GluR2−/−or GluR2+/−) were performed alongside wild-type littermates (GluR2+/+) for controls. Experimental protocols were approved by The Hospital for Sick Children Animal Care Committee.

### Extracellular fEPSP electrophysiological recordings

The preparation of brain slices has been previously described [10], [30]. Briefly, hippocampal slices (400 µm) were obtained from 3 to 6 month old adult mice and allowed to recover in a submerged holding chamber for at least 1 hour. A single slice was then transferred to the recording chamber and submerged and superfused with 95% O_2_-5% CO_2_ saturated artificial cerebral spinal fluid (ACSF, 2 ml/min) at a temperature of 28°C. The ACSF contained (in mM) 120 NaCl, 2.5 KCl, 1.3 MgSO_4_, 1.0 NaH_2_PO_4_, 26 NaHCO_3_, 2.5 CaCl_2_, and 11 D-glucose. For field EPSPs, the recording pipette (3 MΩ) was filled with ACSF solution.

Synaptic responses were evoked by bipolar tungsten electrodes placed 200–400 µm from the cell body layer in the CA1 area. fEPSPs were measured by taking the slope of the rising phase between 5% and 60% of the peak response. All data acquisition and analysis were done using pCLAMP 7 software (Axon instruments). After a stable baseline period, LTP was induced by high-frequency stimulation (HFS) using 2 trains of 100 Hz at 10 second intervals (with each train lasting 1 s) or a TBS protocol (total 60 pulses) while late-phase LTP (L-LTP) was induced by 4 trains of 100 Hz at 20 second intervals (with each train lasting 1 s). Paired-pulse facilitation (PPF) was recorded prior to and following induction of LTP (with the first and second responses being separated by an interval of 50 ms). The ratio of the slope of the second response to the slope of the first response was subsequently calculated. All drugs for field EPSP recordings were purchased from Sigma Aldrich (Oakville, Canada), Tocris (Missouri, U.S.A.) and LC Laboratories (Massachusetts, U.S.A.). D,L-AP5 and IEM-1460 were dissolved in distilled water. Staurosporine, KN-62, PD98059, U0126, LY294002, wortmannin and anisomycin were dissolved in DMSO. For tests of inhibition of LTP, drugs were added to ACSF perfusate 15–20 minutes before the LTP induction protocol was initiated (with staurosporine, KN-62, PD98059, U0126, LY294002, wortmannin and anisomycin having a maximum final concentration of 0.1% DMSO or less after being added to ACSF). For experiments concerning the maintenance phase of L-LTP, PD98059 was added to the ACSF perfusate 20–30 minutes post-induction. Vehicle treatments were performed with 0.1% DMSO and/or distilled water depending on the drug/drugs perfused. All inhibition studies with mutant animals (GluR2−/− or GluR2+/−) were performed alongside wild-type littermates (GluR2+/+) for controls. For comparison of the magnitude of LTP between different groups, the last 5–10 minutes of recordings were compared statistically. n represents the number of hippocampal slices used in each experiment. Normally, one slice per mouse was used for experiments. When average data was plotted, data was normalized to the average of the baseline responses unless indicated otherwise. The representative traces were averages of four successive sweeps during recording. All data was statistically evaluated by Student's *t*-test. Experimental protocols were approved by The Hospital for Sick Children Animal Care Committee.

### Whole-cell voltage-clamp recordings

Acute hippocampal slices of 300 µm thickness were prepared from GluR2 knockout mice and littermates at 20–30 days of age. Slicing and recovery procedures are the same as in fEPSP recordings. Electrodes (3–4 M 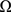) contained (in mM) 120 Cs-methanesulfonate, 5 NaCl, 1 MgCl_2_, 0.5 EGTA, 2 Mg-ATP, 0.1 Na_3_GTP, 20 HEPES, pH 7.2, 280–300 mOsm. Picrotoxin (100 µM) was used in ASCF to eliminate GABAergic transmissions. D,L-AP5 (100 µM) was administrated in perfusate throughout all CP-AMPAR dependent LTP experiments. The internal solution for BAPTA (30 mM) experiments was separately made. For tetanus toxin (TeTx) experiments, stock solution was prepared at 1,000× of final concentration (75 nM, 250 nM) and was added to internal solution just prior to recordings. The same amount of tetanus toxin was boiled for 5 minutes prior and used as inactive control. Test pulses were given at 0.1 Hz and neurons were clamped at −65 mV during basal level recording and LTP recording. Whole-cell mode was briefly switched to current-clamp mode to allow depolarization induced by theta burst stimulation (15 bursts with 200 msec interval of 4 pulses at 100 Hz). A −3 mV step was given 250 msec after each test pulse to monitor membrane and access resistances. Data acquisition and analysis were done using pCLAMP 8. Only recordings with a drift of access and membrane resistances less than 20% were included for statistical analysis by Student's *t*-test. For comparison of the magnitude of LTP between different groups, the last 5 minutes of recordings were compared statistically. n represents the number of hippocampal slices used in each experiment, with normally only one slice per mouse used for experiments. The representative traces were averages of five successive sweeps during recording. All drugs for whole-cell recordings chemicals were purchased from Sigma Aldrich (Oakville, Canada). Tetanus toxin light chain fragment was a generous gift from Dr. William Trimble. Experimental protocols were approved by The Hospital for Sick Children Animal Care Committee.

## Supporting Information

Figure S1 Figure demonstrating that the administration of D,L-AP5 in the ACSF perfusate blocks TBS-LTP in wild-type slices during whole-cell recordings.
(0.05 MB PDF)
