# Supplementary material for: Ca2+ Permeable AMPA Receptor Induced Long-Term Potentiation Requires PI3/MAP Kinases but Not Ca/CaM-Dependent Kinase II
Source: PLoS One. 2009 Feb 3;4(2):e4339. doi: 10.1371/journal.pone.0004339 (PMC2629531; doi:10.1371/journal.pone.0004339)
Supplement: Figure S1 — Figure demonstrating that the administration of D,L-AP5 in the ACSF perfusate blocks TBS-LTP in wild-type slices during whole-cell recordings. (0.05 MB PDF) [file pone.0004339.s001.pdf]

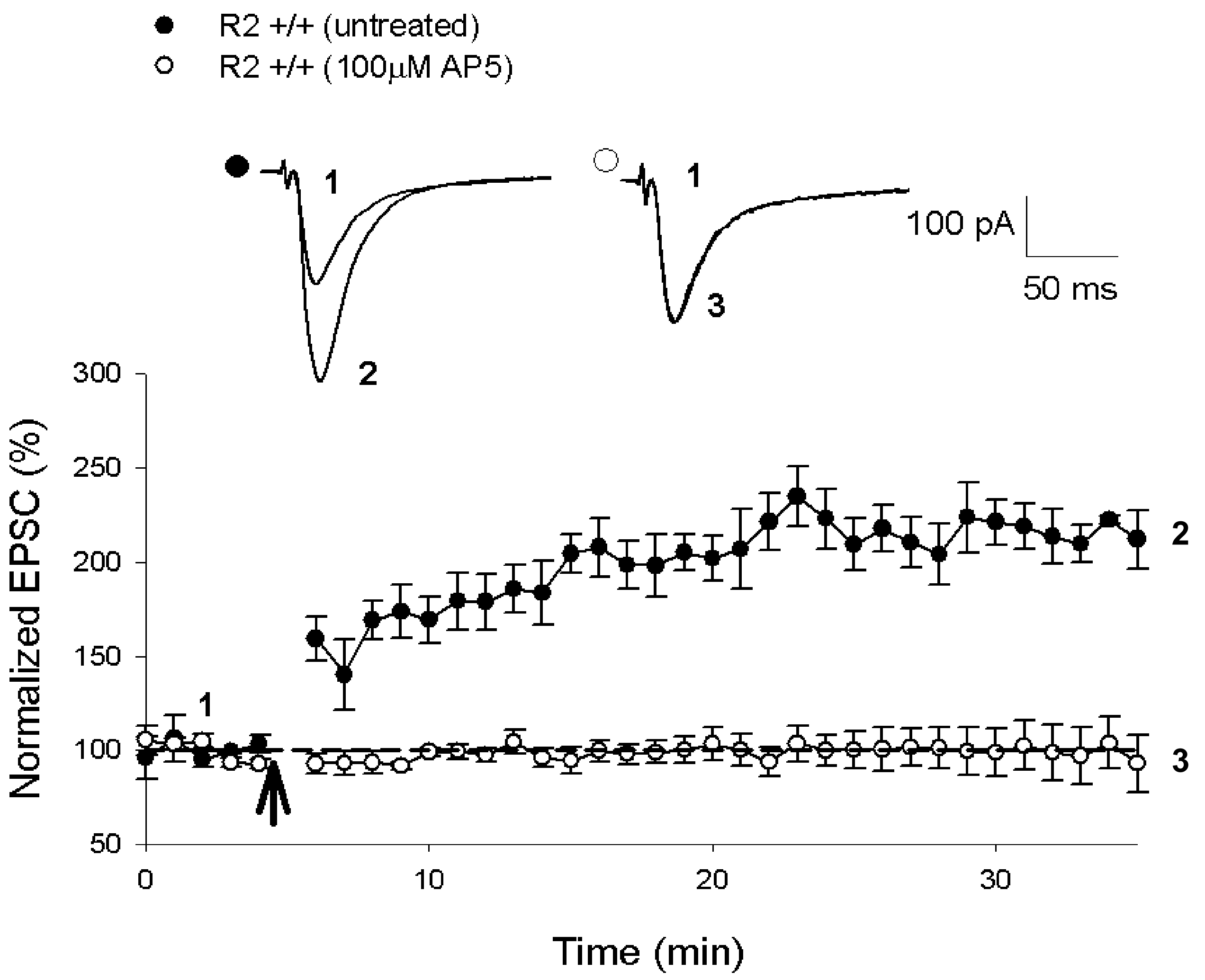

Figure S1. The presence of D,L-AP5 completely inhibited NMDAR-dependent LTP induced in wild-type slices by TBS during whole-cell recordings (control = 217 ± 8.6, n = 5 versus D,L-AP5 treated = 102 ± 14, n = 6; *P* < 0.001).
